# A Scalable FPGA Architecture for Randomly Connected Networks of Hodgkin-Huxley Neurons

**DOI:** 10.3389/fnins.2018.00698

**Published:** 2018-10-09

**Authors:** Kaveh Akbarzadeh-Sherbaf, Behrooz Abdoli, Saeed Safari, Abdol-Hossein Vahabie

**Affiliations:** ^1^High Performance Embedded Architecture Lab., School of Electrical and Computer Engineering, College of Engineering, University of Tehran, Tehran, Iran; ^2^School of Cognitive Sciences, Institute for Research in Fundamental Sciences, Tehran, Iran

**Keywords:** Hodgkin-Huxley model, spiking neural network, scalable hardware architecture, connectivity matrix, approximate computing, permutation matrix

## Abstract

Human intelligence relies on the vast number of neurons and their interconnections that form a parallel computing engine. If we tend to design a brain-like machine, we will have no choice but to employ many spiking neurons, each one has a large number of synapses. Such a neuronal network is not only compute-intensive but also memory-intensive. The performance and the configurability of the modern FPGAs make them suitable hardware solutions to deal with these challenges. This paper presents a scalable architecture to simulate a randomly connected network of Hodgkin-Huxley neurons. To demonstrate that our architecture eliminates the need to use a high-end device, we employ the XC7A200T, a member of the mid-range Xilinx Artix®-7 family, as our target device. A set of techniques are proposed to reduce the memory usage and computational requirements. Here we introduce a multi-core architecture in which each core can update the states of a group of neurons stored in its corresponding memory bank. The proposed system uses a novel method to generate the connectivity vectors on the fly instead of storing them in a huge memory. This technique is based on a cyclic permutation of a single prestored connectivity vector per core. Moreover, to reduce both the resource usage and the computational latency even more, a novel approximate two-level counter is introduced to count the number of the spikes at the synapse for the sparse network. The first level is a low cost saturated counter implemented on FPGA lookup tables that reduces the number of inputs to the second level exact adder tree. It, therefore, results in much lower hardware cost for the counter circuit. These techniques along with pipelining make it possible to have a high-performance, scalable architecture, which could be configured for either a real-time simulation of up to 5120 neurons or a large-scale simulation of up to 65536 neurons in an appropriate execution time on a cost-optimized FPGA.

## 1. Introduction

Computational neuroscientists and computer researchers have two complementary views on mathematical modeling of the human intelligence. While the neuroscientists try to find the mathematical models of experimental results, computer scientists, due to the limited processing power, attempt to develop intelligent algorithms just inspired by the neuroscientific theories. There is generally a gap between the algorithms developed by these two groups from the intelligent point of view. Furthermore, the advent of the deep learning makes this gap even wider. The outstanding success of deep learning is deeply indebted to the emergent software libraries (Jia et al., 2014; Abadi et al., 2015; Seide and Agarwal, 2016) that have employed the power of graphical processing units (GPUs) and the hardware frameworks in both application specific integrated circuits (ASICs) (Jouppi et al., 2017) and field programmable gate arrays (FPGAs) (Caulfield et al., 2016; Mahajan et al., 2016).

Spiking neural networks (SNNs) are more complicated than artificial neural networks (ANNs) in terms of neuron models and the number of synapses. While neuron model of ANN employs the multiplication and addition of weights and inputs followed by a linear or nonlinear activation function, the spiking neuron models such as Izhikevich simple model (Izhikevich, 2003), FitzHugh-Nagumo (Izhikevich, 2007), and Hodgkin-Huxley (Hodgkin and Huxley, 1952) are continuous nonlinear dynamical systems. Implementing a continuous model in a digital computing machine is done by discretizing the model which results in more arithmetic operations. According to the type of neuron model, it may be far more compute-intensive than neuron models used in ANNs. If we define the number of synapses (aka weights in the ANN literature) as a metric for the size of a network, while the size of a large DNN is about a few millions, for the brain it is about 100e6 times larger (Gerstner et al., 2014). In fact, the brain relies on the parallelism instead of fast neuronal cells. A variety of software frameworks have been introduced to model a single neuron or a network of neurons in detail. NEURON (Carnevale and Hines, 2006), GENESIS (Kornbaum and Enderle, 1995), NEST (Gewaltig and Diesmann, 2007), Brian (Goodman and Brette, 2009), NeMo (Fidjeland et al., 2009), PCSIM (Pecevski, 2009), PyNN (Davison, 2009), and HRLSim (Minkovich et al., 2014) are of the most cited ones. Although all of these frameworks today support GPUs to speed up simulations (Brette et al., 2007), their hardwired micro-architectures put obstacles in the way of parallelism utilization for irregular applications such as spiking neural networks (Fung, 2015). A custom hardware design using either ASIC or FPGA is an alternative way that is more amenable to parallelism.

When it comes to talking about ASIC, we have three ways ahead. It is possible to design a circuit in a fully digital, fully analog, or mixed-signal manner. Some projects employ the digital way to implement their circuit. A digital circuit is easy to design and fabricate. Nevertheless, it consumes more area and more power than the analog ones. Some other projects utilize the analog circuit to reduce the power consumption and achieve more neuron density. Meanwhile, they use a digital circuit to provide a high-speed communication environment.

SpiNNaker (Furber et al., 2013) is the world's biggest network on chip (NOC) powered by over a million ARM processor cores for real-time simulation of SNNs. IBM TrueNorth (Merolla et al., 2014) is another major project that utilizes a fully digital approach to realize a million leaky integrate and fire (LIF) neurons with 256 synapses per neuron. Their proposed architecture is scalable which means that it is possible to enlarge the network as needed. Neurogrid (Benjamin et al., 2014) is a project that has chosen the mixed signal manner to design the system. One setup of this system using 16 Neurocores has made it possible to simulate one million neurons with a billion synapses in real-time. BrainScaleS is the worthy successor of FACET project (Schemmel et al., 2010). This European project has two major outcomes. In the first step, they developed a unified software framework called PyNN that makes it simple to verify the accuracy of their hardware by running the desired model on either NEST or their chip. In the second step, which is the main aim of this project, they have designed a wafer-scale mixed signal circuit. While the dynamics of adaptive exponential integrate and fire (AdEx) neuron model and synapses are implemented in the analog part of the circuit, digital circuits are mainly used in the inter-wafer communication. There is a growing demand for scaling up the size and complexity of the hardwares designed for simulating SNNs. Trying to address this need, researchers have employed emerging technologies such as memristors.

Memristive devices can be used as alternative solutions to implement synapses. For instance, in Azghadi et al. (2017), a hybrid CMOS-memristor circuit is proposed to mimic some essential learning features of biological synapses. Their proposed circuit is almost ten times smaller than its pure CMOS equivalents. Using memristors in a crossbar array structure delivers a nanoscale, low power, and compact circuit (Hu et al., 2017). Moreover, memristors are easily tunable for plasticity rules which give rise to a compact implementation of spike time dependent plasticity (STDP) and spike rate dependent plasticity (SRDP) (Bill Legenstein, 2014). To demonstrate the application of memristors in machine learning, a memristor based crossbar structure is proposed for handwritten digits recognition with a recognition accuracy of 97.10% for MNIST benchmark (Zheng and Mazumder, 2018).

Unfortunately, despite all these efforts, the ASIC suffers from inflexibility that means neuron models, synapses, and synaptic plasticity algorithms are hardwired into the silicon and it is not possible to adapt them to other models. As FPGAs are today common among companies such as Microsoft (Caulfield et al., 2016) and Baidu (Ouyang et al., 2014) for the deep learning applications, their flexibility opens up a good opportunity for reconfigurable SNNs' accelerators.

Some research projects exploit FPGAs in the heart of their SNN. For example, in Yaghini Bonabi et al. (2014), the network consists of two competitive mini-columns of HH neuron model. The final design is implemented on a Xilinx Virtex 7 FPGA. Another example is the work reported in Pani et al. (2017). They utilize Xilinx Virtex 6 to implement a 1440 fully connected network using Izhikevic neuron model. Their proposed architecture is able to run the simulation in real-time at a sampling rate of 10 kHz. Some researchers focus on specific applications such as (Yang et al., 2018) that presents an FPGA implementation of a real-time scalable hardware platform for a hippocampal spiking neural network. They employ a randomly connected SNN with a 10K Izhikevich neuron model which is organized in four excitatory neuron groups (NGs) and one inhibitory NG with sparse synaptic connections. Each NG is accompanied by one synaptic group (SG) that calculates input synaptic currents to the NGs. In a similar context, (Yang et al., 2017) introduces multiplier-less techniques to implement a digital SNN for a conductance-based subthalamic nucleus model. Their techniques consist of a novel “shift MUL” method and piecewise linear approximation. From the perspective of generality, NeuroFlow (Cheung et al., 2016) is a general purpose simulator which has been exclusively developed for a cluster of FPGAs provided by Maxeler Technologies. This generality is achieved by high-level synthesis (HLS) provided by the tools from the Maxeler called MaxCompiler. This compiler receives a java code describing the behavior of the network and generates an equivalent register transfer level (RTL) code in a hardware description language that is either Verilog or VHDL. HLS is relatively a new trend in digital design which aims to reduce the development time, but it usually infers an inefficient hardware in terms of area and performance. HLS has some major limitations that cause some difficulties from hardware reuse, data types, and level of parallelism points of views.

This brief review highlights a gap in the hardware implementations of SNNs; that is, the need to give more attention to FPGA implementations. In this work, we are going to propose a scalable architecture, which can be configured as a real-time or a large-scale randomly (or fully) connected network, or something in between. To reach this goal, we propose a hardware module which reads states of neurons in succession, calculates the synaptic efficacy on the corresponding neuron, updates its state, and writes it back to its memory bank. This core processes the neuron states serially. To improve performance through parallelism, we employ multiple excitatory or inhibitory cores. Updating each synaptic conductance requires sharing firing activities among the cores, this is performed by a vector which is responsible for storing the spike occurrence per iteration per neuron. Moreover, a detailed exploration of our neuronal network model with different number system representations leads us to select the Q9.24 number format as a trade-off between the accuracy and performance. Although this architecture has been accompanied by several techniques to improve the accuracy and performance, our major contribution is introducing a novel exact/approximate method to update synaptic conductances (section 2.6.2). This approach shows a great potential to create embedded systems for randomly connected networks of any neuron models using FPGAs.

In section 2, we introduce the neuron and the network model and discuss the details of the number system representations. Then, we examine how linear approximation reduces the hardware cost. At the end of this section, we present both single cycle and 7-stage pipeline implementations of our proposed architecture. In section 3, we show the accuracy of our implementation by comparing the hardware and the software results. In addition, we report the effects of the memory configurations on the performance and the number of neurons. Finally, section 4 belongs to discussion and conclusion.

## 2. Materials and methods

### 2.1. Neuron and network model

**Figure 1:**
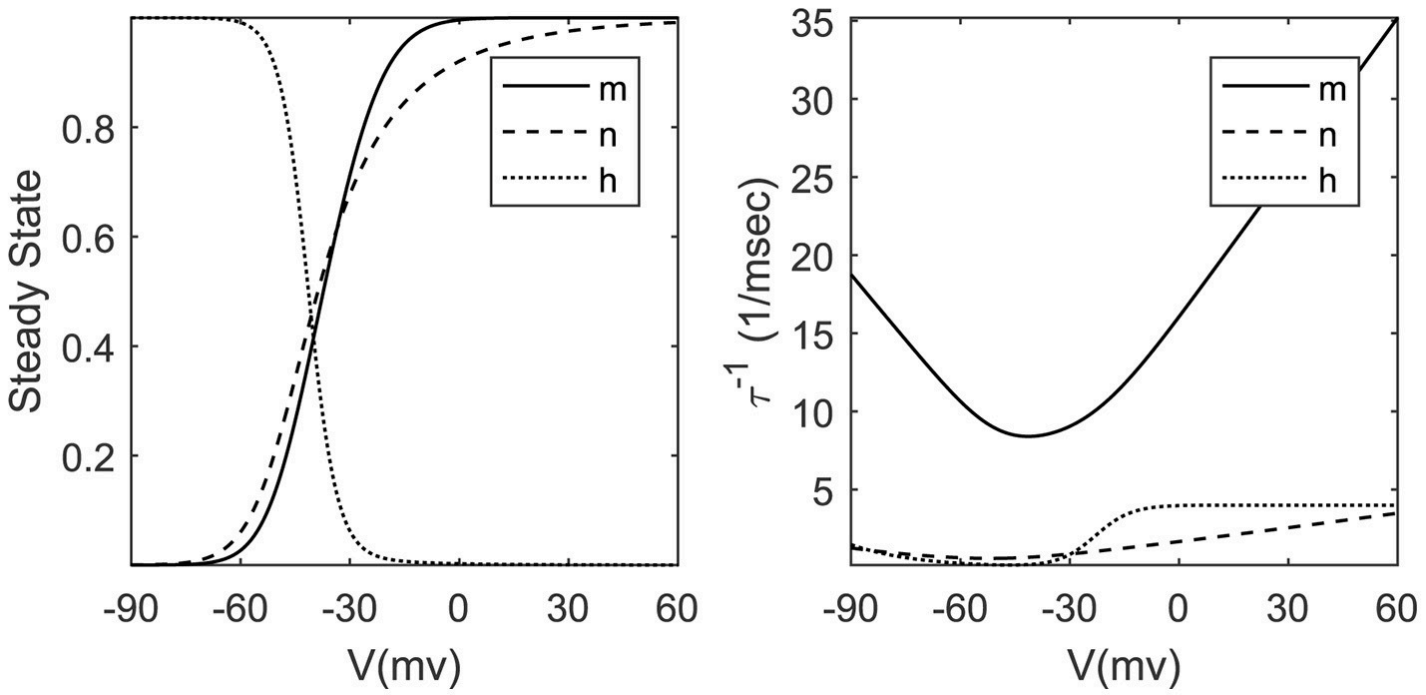
Steady-state values and inverse time constants of gating variables.

In this section, we present the original Hodgkin-Huxley neuron model and the structure of the neuronal network used by this paper. The HH model comprises both transient and persistent currents of Na and K ions as well as an overall leak current that indicates the impact of all other ionic channels (Izhikevich, 2007). The standard complete set of equations consists of a first order differential equation depicting membrane voltage and three other equations to describe gating variables; that are,

(1) $$\begin{array}{r} C_{m}\frac{\text{d}v}{\text{d}t}=I_{ext}-{\bar{g}}_{Na}m^{3}h(v-E_{Na})-{\bar{g}}_{K}n^{4}(v-E_{K})-{\bar{g}}_{L}(v-E_{L}),\\ \frac{\text{dx}}{\text{d}t}=\alpha_{x}(v)(1-x)+\beta_{x}(v)x=\frac{\left(x_{\infty }(v)-x\right)}{\tau_{x}(v)}, \end{array}$$

that *x* stands for *m*, *n*, *h*; and,

(2) $$x_{\infty }(v)=\frac{\alpha_{x}(v)}{\alpha_{x}(v)+\beta_{x}(v)},\tau_{x}(v)=\frac{1}{\alpha_{x}(v)+\beta_{x}(v)},$$

where the parameters alpha and beta are transition rates between open and closed states of related ionic channels. For the sake of simplicity, we can ignore the detailed description of alpha and beta equations and approximate the time constant τ_*x*_ and steady-state value of gating variable *x*_∞_ by Boltzmann and Gaussian functions (Dayan and Abbott, 2005; Ermentrout and Terman, 2010) stated in Figure 1. The exponential nature of these voltage-dependent variables poses a serious challenge to hardware designers. Implementing exponential functions is costly from the logic resources point of view; therefore we look for approximate methods with lower cost. In section 2.4, we will explain a piecewise linear technique to approximate *x*_∞_ and $\tau_{x}^{-1}$. Approximating inverse time constant eliminates the need for a lot of division hardware that is one of the most resource consuming elementary operators in the hardware designer community.

There are various types of network topologies such as small world (Yu et al., 2011, 2013) and randomly connected (Brette et al., 2007). Since we are interested in reservoir computing paradigm, we select a suitable topology for this context. Rich dynamics of randomly connected networks have been increasingly utilized in the context of reservoir computing paradigm (Lukoševičius and Jaeger, 2009). In this paper, therefore, an architecture is proposed to speed up the COBAHH (Hodgkin-Huxley neuron model with conductance-based synapses) network model introduced in Brette et al. (2007). This network uses a randomly connected network of the HH neuron model with exponential synaptic conductance. Embedding the synapses dynamics in Equation (1) is performed by replacing *I*_*ext*_ with appropriate expressions for inhibitory and excitatory connections. The resulting expression,

(3) $$\begin{array}{r} I_{ext}=-g_{e}(v-E_{e})-g_{i}(v-E_{i}),\\ \frac{\text{d}g_{e}}{\text{d}t}=\frac{g_{e}}{\tau_{e}},\\ \frac{\text{d}g_{i}}{\text{d}t}=\frac{g_{i}}{\tau_{i}}. \end{array}$$

has two extra terms to mimic the behavior of excitatory and inhibitory synaptic channels. *g*_*e*_ and *g*_*i*_ are the excitatory and inhibitory conductances respectively. When an inhibitory or an excitatory neuron fires, fixed weights (*w*_*i*_ and *w*_*e*_) will be added to the corresponding conductances (*g*_*i*_ and *g*_*e*_) of its post synaptic neurons, regardless of which neuron fires. More details about the network model and Python implementation are available in the Brian simulator (Stimberg et al., 2014). We choose this network model due to its generality. We can use a hardware design that describes this network to model any randomly connected network. The major limitation of this network is its fixed inhibitory and excitatory weights.

Implementing such a network in hardware is strongly challenged by the number system representation and the target hardware platform. In the next two sections, we try to answer to following two questions:

1. What are the effects of number system representation on the accuracy and performance?
2. What constraints does target FPGA impose on our design?

### 2.2. Number system representation

There are two main binary number system representations called floating-point and fixed-point. From the hardware implementation point of view, while the first one consumes a high amount of logic resources, the second one is hardware friendly. Therefore, when we are going to design a digital hardware, the first question that comes to mind is the number system representation and the number of bits required to uphold the precision. In this subsection, our main aim is to determine how fixed-point and floating-point representations and their bit width affect accuracy.

Dynamic range and the gap between two consecutive numbers are the most important distinguishable criteria (Ercegovac and Lang, 2003). The floating-point number system has a larger dynamic range in contrast to the fixed-point number system, which means that the corresponding range of values in floating-point numbers are wider than fixed-point numbers for the same word length. To determine the required dynamic range, we should extract the upper and lower bounds of each variable using a software model. The simulation results show that an intermediate variable has the greatest dynamic range. It is noticeable that this variable almost varies from -90 to 130. Hence we require either nine or eight integer bits to represent all variables in unbiased or biased representations respectively. A biased representation requires a bias compensation method to produce true results. The compensation methods cost a lot from the hardware resource requirements point of view (Ercegovac and Lang, 2003). In order to reduce the hardware cost, we choose an unbiased representation with nine integer bits. Consequently, although the large dynamic range is a definite advantage of the floating-point representation, simulation results show that this feature does not justify heavy hardware implementation cost.

**Figure 2:**
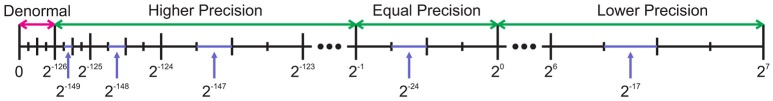
Comparing gaps between two adjacent numbers for a single-precision floating-point number and a 32-bit fixed-point number. The fixed-point number has 24 fractional bits. The positive side is displayed. While the single-precision number has higher or equal precision for values below one, it shows lower precision for other values.

**Figure 3:**
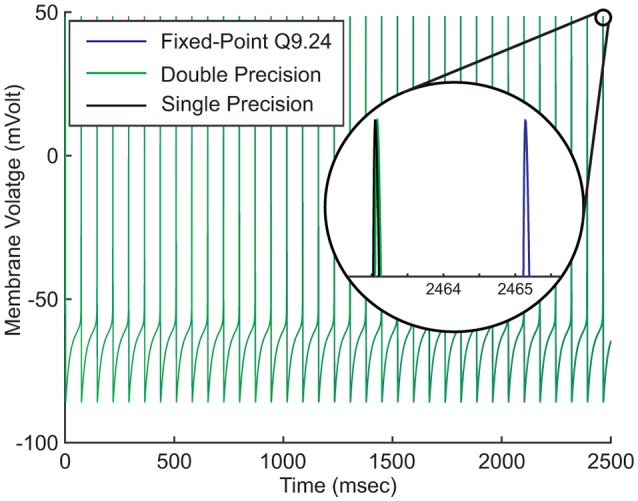
Effects of number system representations on the accuracy of the model. Hodgkin-Huxley model is described as a continuous dynamical system. Digital realization of the system requires discretizing both value and time. Discretization introduces round-off errors to digital systems. Higher round-off errors result in more time shift.

The gap between two adjacent numbers is a criterion for measuring precision. To investigate the effects of this gap on the accuracy of our model, firstly, we should study the floating-point and fixed-point representations in more details. The IEEE-754-1985 introduces two commonly used formats for floating-point computation: the 32-bit single-precision and the 64-bit double-precision. There are not any standards for fixed-point numbers, so its precision only depends on the designer's decision about the number of fractional bits. While the fixed-point system presents a constant gap between any two consecutive numbers, the floating-point system exhibits different gaps for different pairs. For the sake of comparison, we compare the single-precision floating-point number with the 32-bit fixed-point number (Q8.24). We suppose that this number of integer bits is the minimum number required to cover the dynamic range of all variables. The value of each number in the single-precision format is *F* = −1^*s*^1.*f*2^*e*−127^ where *e* varies from 1 to 254 for normal values. The smallest positive normal value is 2^−126^ that is achieved by selecting *s* = 0, *f* = 0, *e* = 1. This value is 2^102^ times smaller than the smallest positive value of the fixed-point counterpart that is 2^−24^. This difference gets even worse for denormal values, but that is not our point. The gap between any pairs of two consecutive numbers in the fixed-point system is 2^−24^ but the story for the floating-point numbers is different. Figure 2 shows the positive side of the variable range. As it can be seen, as the exponent increments, the gap increases. Consequently, while the floating-point numbers exhibit higher precision than the fixed-point equivalents for the values below 0.5, they show lower precision for the numbers greater than 1. This behavior is more complicated than what can be easily analyzed. More simulations are required to scrutinize the effect of the number system precision on our neuron model, as shown in Figure 3.

Figure 3 compares the fixed-point (Q9.24) and the floating-point (single and double precision) implementations of our neuron model. The neuron's parameters are set in such a way that it fires regularly without external stimulus. We run this simulation for just about 2500ms. Magnifying the figure reveals that the spike time differences are negligible at the start of the simulation. As the simulation proceeds, the later spikes show small displacements that increase under time evolution. As a result of discretization, there is not an ultimate precision. There are always spike time differences, regardless of the number system representation or the number of bits.

The result of a computation is not usually an exact number. If we call two adjacent numbers in the digital arithmetic *F*1 and *F*2 and the exact result *x*, then *F*1 ≤ *x* ≤ *F*2. Rounding is the process of assigning one of *F*1 or *F*2 to *x*. The roundoff error is the difference between x and its assigned value. Different roundoff errors result in the appearance of spikes at the different times.

Due to its lower hardware cost, in this paper, we use the fixed-point number representation system. As we said above, we require 9 bits for the integer part. The number of fractional bits is a trade-off between accuracy and resource usage, which will be discussed in section 2.5. This leads us to have not only a compact but also an accurate enough circuit in comparison with the floating-point implementation.

### 2.3. Hardware platform constraints

The second question is about the hardware platform. In this paper, we use the Nexys Video trainer board from Digilent. Digilent has employed a XC7A200T device, the most powerful member of cost-optimized Artix®-7 family, in this board. We choose this board as our processing engine to show the possibility of fast simulation of relatively large-scale SNN with a low cost embedded solution. Xilinx combines traditional reconfigurable logic gates with on-chip memories, digital signal processing (DSP) blocks, and gigabit transceivers in XC7A200T. The variety of additional blocks available on FPGAs makes them perfectly suitable for digital reconfigurable tasks such as SNNs.

Although there are essential add-on blocks in the Artix FPGAs, they have some inherent limitations. The finite amount of memory cells that are available in a FPGA is a major limitation. The memory limitation results in fewer locations to store the neuron states and the connectivity matrix. In addition, operand sizes of the DSP blocks are limited to what are offered by the manufacturer. In particular, DSP48E1, which is the code name for the DSP blocks used in 7-series FPGA of Xilinx company, contains two's complement multipliers that support only operand widths up to 25 bits. Regarding the target device, we may require more than one DSP block per operation. Given these issues, in the following, we propose some methods to better utilize these valuable resources.

### 2.4. Piecewise linear approximation

As mentioned in section 2.1, time constants and steady-state values of gating variables are approximated by Boltzmann and Gaussian functions. Implementing any nonlinear function via logical gates requires algorithms to describe them by basic elementary arithmetic operators. Polynomial approximation using techniques such as Taylor expansion and *n*^*th*^ order spline are commonly used in the literature to approximate arbitrary functions including exponential ones (Jupp, 1978; Miyata and Shen, 2005). Although all of them show reasonable accuracy under certain circumstances, their complexities make it difficult to have a small and fast hardware implementation. Iterative techniques such as CORDIC approach reduce the hardware size, but its iterative nature causes more latency than the noniterative algorithms (Parhami, 2010; Yaghini Bonabi et al., 2014).

**Figure 4:**
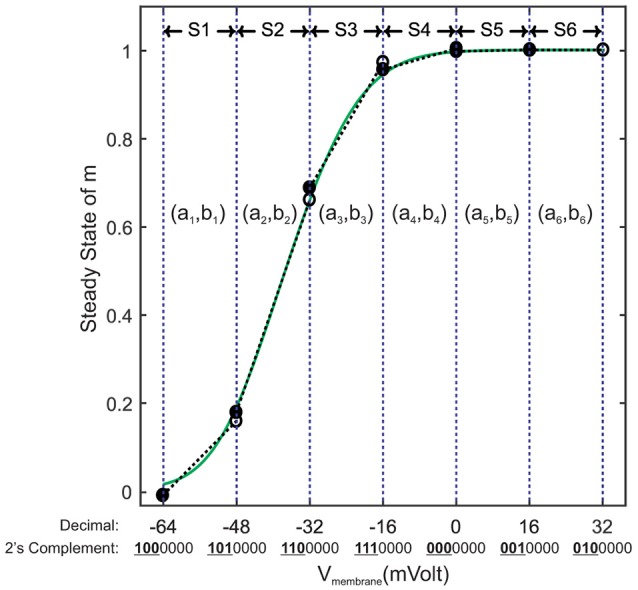
An example of linearization for *m*_*inf*_. Its range is split into six equal segments called S1 to S6 with step size of 16*mV*. First three left bits of each segment's lower endpoint indicate the location of the memory address used to store the slope and y-intercept of the corresponding straight line (*a*_*k*_, *b*_*k*_).

Piecewise linear (PWL) approximation can exhibit low resource consumption as well as low latency. Meanwhile, it can show high accuracy with well-tuned parameters. There are fast and accurate PWL techniques especially for approximating the sigmoid functions. These techniques are widely used to estimate the activation function of ANNs (Tommiska, 2003; Basterretxea et al., 2004; Lin et al., 2013). In this paper, we partition the entire domain into equal intervals. The step size is equal to 2^*k*^, *k* ∈ *Z* to reduce the bit width of the memory locations used to store the PWL coefficients. For each segment, we extract a line equation in the slope-intercept form (*y* = *ax*+*b*) using the least square algorithm (Apostol, 1969), then we store the values of slope *a* and y-intercept *b* in a memory row. Figure 4 shows a demo of this linearization technique. As it can be seen, some memory locations are not available in the address table. This leads us to have a smaller area if we use distributed memories instead of block ones. This figure illustrates another matter of importance, two segments that are on the right-hand side (S5 and S6) demonstrate the same slope and y-intercept. This fact makes it possible to combine them as a single super segment to reduce the memory size. Super segments may appear at any intervals. Fortunately, synthesis tool automatically locates all super segments and stores them each in a single memory row.

### 2.5. FPGA implementation of a single neuron

**Figure 5:**
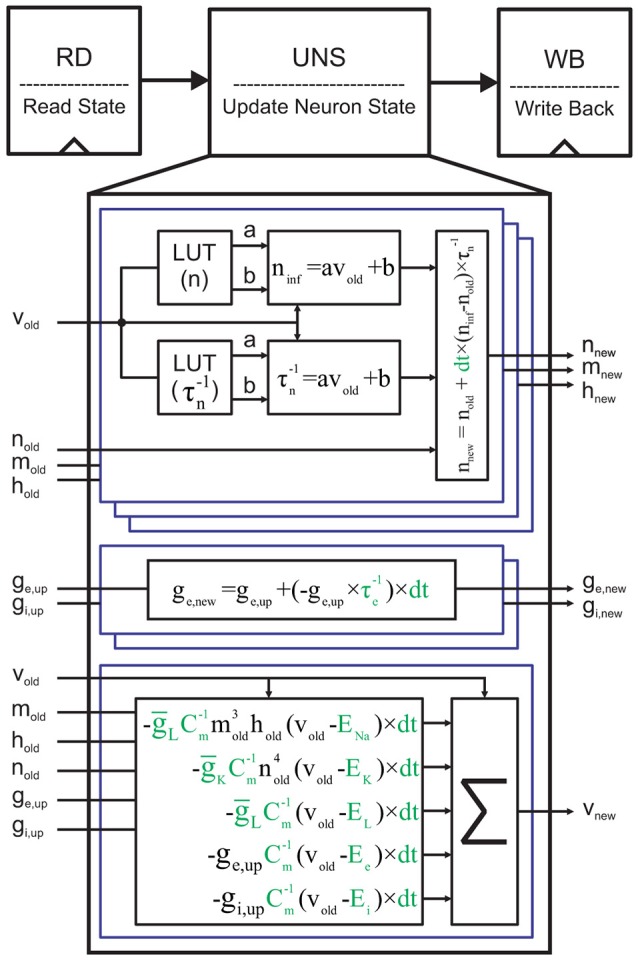
Single cycle implementation of a single neuron. Reading the current neuron state, updating it, and writing the new state back to the memory are the three stages. The *g*_*e,up*_ and *g*_*i,up*_ are the updated versions of the old synaptic conductances (*g*_*e,old*_, and *g*_*i,old*_) with regard to the presynaptic neuronal activities. It should be noted that the corresponding memory locations of *g*_*e,old*_ and *g*_*i,old*_ are filled with zeros for the single neuron implementation. Moreover, since there is not any presynaptic neurons, *g*_*e,up*_ = *g*_*e,old*_ and *g*_*i,up*_ = *g*_*i,old*_. For the sake of clarity, the constants are displayed in green.

To implement a single neuron on FPGA using the Euler method, we employ a simple 3-stage pipeline (Figure 5). In the first stage, we read (RD) the current state of neuronal variables, called here *v*_*old*_, *n*_*old*_, *m*_*old*_, *h*_*old*_, *g*_*e,old*_, and *g*_*i,old*_, from a memory address. In the next stage, we compute the next state values of these variables using the *update neuron state* (UNS) block. Finally, the updated state is written back to the same memory address in the *write back* (WB) stage. UNS is the most compute-intensive block. It contains six sub-blocks to compute the new states of the gating variables, the synaptic conductance, and the membrane voltage depicted in Equation (**3**). Each gating variable requires two lookup tables (LUTs) to hold the slope and the y-intercept of its voltage-dependent steady-state value and time constant. An arithmetic block uses the output data of both LUTs to compute the approximate values of *x*_∞_ and $\tau_{x}^{-1}$. We lastly generate *x*_*new*_ using *x*_∞_, $\tau_{x}^{-1}$, and *x*_*old*_. Full details of discretized equations and their parameters are visually available in Figure 5. As we said above, we extract the constants except *dt* from the Brette et al. (2007). In this paper, we suppose that $dt=\frac{1}{128}$ms. This value is a power of two (2^−7^) that converts each division to a simple shift operation. Moreover, this value is small enough to guarantee convergence and accuracy of the Euler method for the neuronal networks with complex neuron dynamics, such as our network model.

We are now ready to finalize our single neuron implementation by filling the LUTs in Figure 5 with the slope and the y-intercept values of the *x*_∞_ and $\tau_{x}^{-1}$ PWL approximations. There remain two parameters still to be determined: (1) The step size (the piece length) in PWL approximations and (2) the required number of fractional bits for the whole architecture including the slope and the y-intercept values. For the sake of simplicity, we write a simple GUI in MATLAB that automatically generates all required synthesizable Verilog files. It only needs an arbitrary step size and a number for fractional bits to produce all necessary files.

As we mentioned in section 2.4, we use equal intervals in this paper. The step sizes for six variables should be selected in such a way that yields errors of the same order of magnitude. Simulation results show that this condition will be fulfilled if the variables are chosen in a way that satisfies $\delta_{m}=\delta_{n}=\delta_{\tau_{n}^{-1}}=2\delta_{h}=4\delta_{\tau_{h}^{-1}}=4\delta_{\tau_{m}^{-1}}$. Here, the symbol δ is a notation for the step size. To analyze the effects of the step size on the error and the resource usage, we initially choose three different step sizes with this constraint. Afterwards, we simulate the PWL models to extract the related errors. Table 1 summarizes the simulation results of MATLAB environment with double precision. As expected, while halving the step size reduces the error, doubling it increases the error a few times larger. The last two columns of Table 1 report the resource consumption of PWL approximations for the Q9.24 number format. Reasons for selecting this format will be discussed in the following paragraphs.

**Table 1:** Linear approximations for the steady-state values and the time constants of the gating variables.

	**Step size name**	**Step size value (δ_*x*_(*mV*))**	**Max Error MATLAB (1e-4)**	**LUTs**	**DSP blocks**
*m*_∞_	Double	4	23.49	74	2
	Base	2	5.9610	69	2
	Half	1	1.4936	159	2
*n*_∞_	Double	4	18.881	70	2
	Base	2	4.7894	67	2
	Half	1	1.1999	165	2
*h*_∞_	Double	2	20.2161	73	2
	Base	1	5.1042	159	2
	Half	0.5	1.2770	240	2
$\tau_{m}^{-1}$	Double	1	11.8597	205	2
	Base	0.5	2.9692	158	2
	Half	0.25	0.80312	295	2
$\tau_{n}^{-1}$	Double	4	17.8569	74	2
	Base	2	4.5093	70	2
	Half	1	1.1532	181	2
$\tau_{h}^{-1}$	Double	1	12.8224	191	2
	Base	0.5	3.2051	157	2
	Half	0.25	0.80196	241	2

*Different step sizes result in different maximum absolute errors for software simulations and resource usages for hardware implementation with Q9.24 number format. The step sizes in the middle rows are called the base, the upper and lower rows show the double and the half step sizes respectively*.

**Figure 6:**
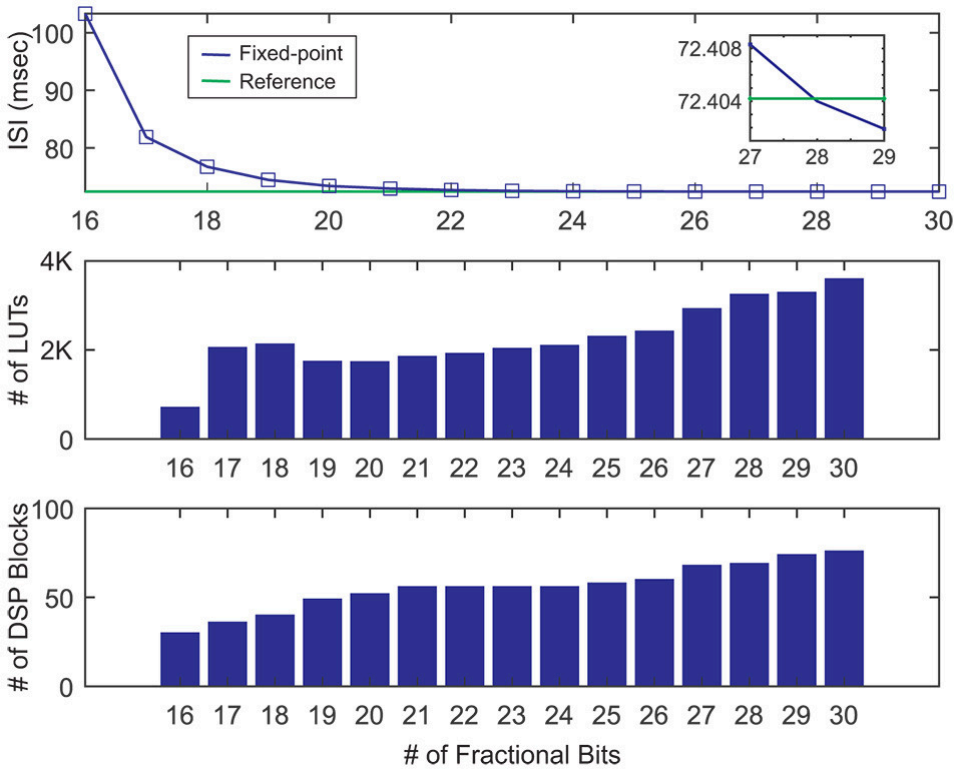
Inter spike intervals and resources usage for various numbers of fractional bits in the fixed-point number representation. Here, we choose the base step size for simulation and sythesis of the single neuron. The reference line (green) shows the ISI of the floating-point representation.

To determine the two above-mentioned parameters, we choose the base step size to simulate and synthesize our single neuron design using VIVADO. Then, we vary the number of fractional bits from 16 to 30. We start from 16 bits simply because there is not tonic spiking behavior below it. As a metric of accuracy, we use the interspike interval (ISI) of our neuron model in the tonic spike regime. Figure 6 (top) shows the ISI of fixed-point representations for a different number of fractional bits in contrast with the ISI calculated by the double precision floating-point format called here the reference ISI. As it can be seen, as the number of bits increases, the ISI gets closer to the reference. Interestingly, the value of ISI gets even smaller than the reference. This is another evidence suggesting that there is no ultimate precision in the digital era. Indeed, selecting the number of fractional bits is a tradeoff between accuracy and resource consumption.

Using the utilization report of VIVADO, we draw the DSP blocks and the LUTs usages in Figure 6 middle, bottom. As these bar graphs indicate, the number of worthy DSP blocks remains constant from 21 to 24 fractional bits. Moreover, the number of LUTs shows a negligible rise. Since the *ISI*_*ref*_ = 72.4042ms and *ISI*_*Q*9.24_ = 72.4651ms, the relative error is $\eta =|\frac{{ISI}_{ref}-{ISI}_{Q9.24}}{{ISI}_{ref}}|\times 100=0.08\%$. Consequently, selecting the Q9.24 format results in a small relative error and low usage of resources. To determine the effect of the step size on the results, we repeat the single neuron simulation for double and half step sizes. There is a close correlation between the number of fractional bits and the step size. If we continuously decrease the step size while the number of fractional bits is fix, we will finally find that the relative error will not be further reduced. According to Table 2, while doubling the step size increases the relative error to almost 25%, halving it has an insignificant effect. Moreover, the resource consumption of the half step size is about 35% larger than base step size. These comments reveal that the base step size is the best choice. In the rest of this paper, we use the base step size to approximate the gating variables and utilize the Q9.24 fixed-point system in arithmetic blocks.

**Table 2:** The inter spike interval (ISI) and the resource usage of the single neuron model in the Q9.24 format for three different step sizes.

**Step size**	**ISI(ms)**	**η(*%*)**	**LUTs**	**DSP blocks**
Double	54.2612	25.05	1711	56
Base	72.4651	0.08	2103	56
Half	72.4676	0.08	2834	56

### 2.6. Proposed pipeline network architecture

**Figure 7:**
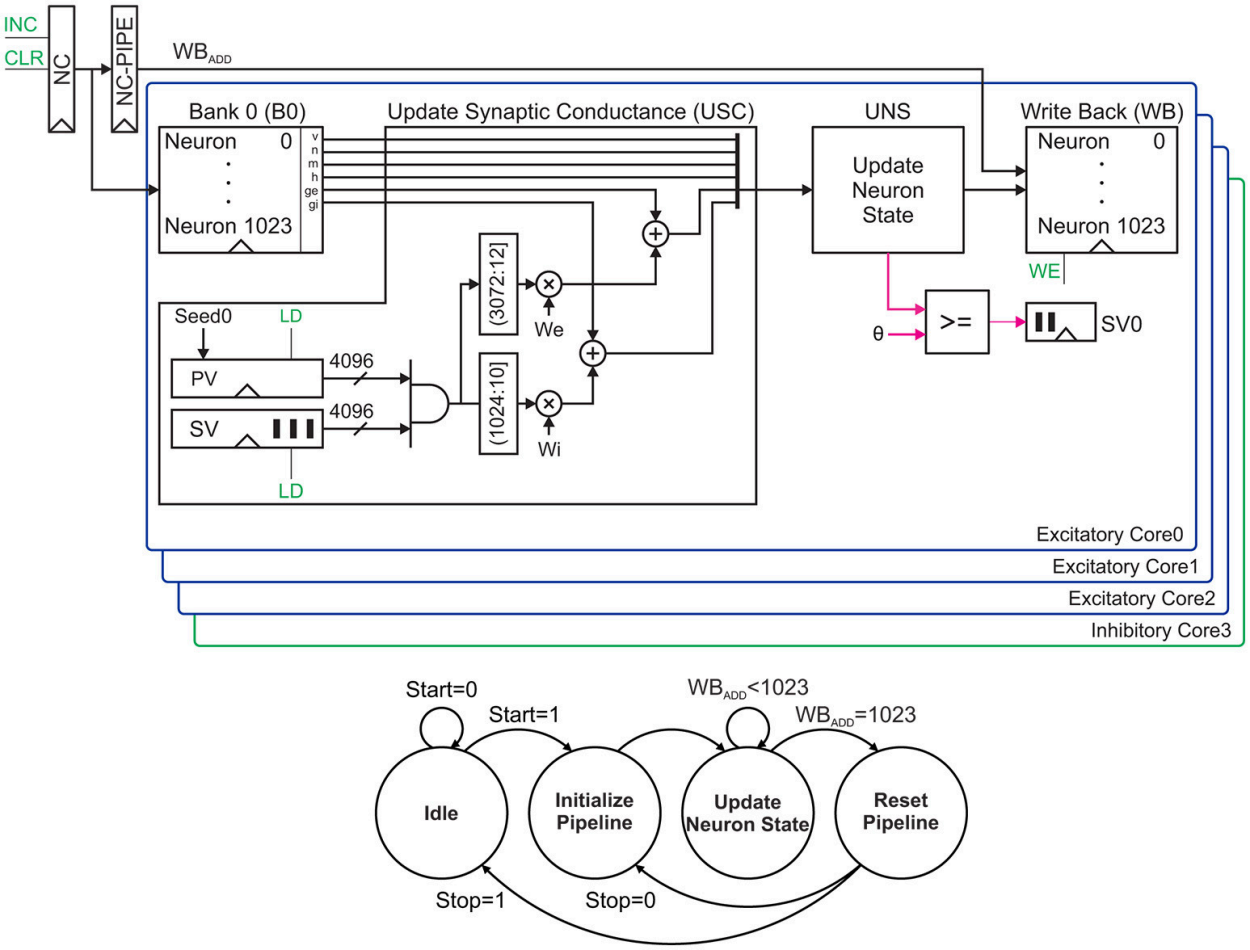
A network architecture that supports 4096 neurons **(Top)** and its finite state machine **(Bottom)**. This architecture consists of four parallel cores for excitatory and inhibitory neurons. Control signals are shown in green. The process starts and terminates by pressing the Start and Stop push buttons respectively.

In this section, we introduce the key concepts of our proposed pipeline architecture. For the sake of clarity, we explain the main idea with an example. Figure 7 **(Top)** illustrates the example of our proposed network architecture. It is often assumed that 20% of neurons are inhibitory and 80% of neurons are excitatory, but our sample network consists of four cores. In order to control the excitation-inhibition balance, we can partially fill the cores. Here, our aim is to validate the functionality. Therefore, we fully fill all the cores. Each core is capable of updating neurons' states in both parallel and serial. The first three cores are excitatory and the last one is inhibitory. Four neuron states are updated in each clock cycle. Meanwhile, each core is responsible for computing next states of 2^10^ = 1024 neurons serially.

The process starts by fetching the states of four neurons from the related memory banks in parallel and then updating their synaptic conductances using the *update synaptic conductance* (USC) units. The UNS units are employed to compute the neurons' next states. The UNS is the same as the unit described in section 2.5 for a single neuron. Each core uses a small comparator to check the membrane voltage for the probable spike. The one-bit output of this comparator is shifted to a 1024-bit first-in, first-out (FIFO) buffer. This FIFO is responsible for storing the spiking activity in each iteration. Finally, the updated states are written back to the memory banks. The memory banks' addresses are generated using a neuron counter (NC). The generated address is propagated by the NC-PIPE to keep the address for the *write back* stage. Controlling this datapath requires a simple finite state machine stated in Figure 7 **(Bottom)**. In following subsections, we will discuss the matter in more details.

#### 2.6.1. Memory banks (neuron state memory)

In this paper, we use block memories to store the states of the neurons. Xilinx 7 series FPGAs have block memories that support Simple Dual-Port mode. This means that each block memory provides two ports, one for writing to the memory and one for reading from it simultaneously. Each block memory can store up to 36Kbits of data and can be configured as a 32K×1, 16K×2, 8K×4, 4K×9, 2K×18, 1K×36, or 512×72 memory. This means that we can save the states of 512 up to 32K neurons on a bunch of block memories without waste of resources. Indeed, this helps us to combine the parallel and serial computing in our architecture. We could increase the degree of parallelism to speed up the simulation using shallower block memories. Furthermore, it is possible to simulate a larger network by increasing the degree of serialization using deeper block memories. We can control the parallelism and serialization by configuring the number and the depth of memory banks.

**Figure 8:**
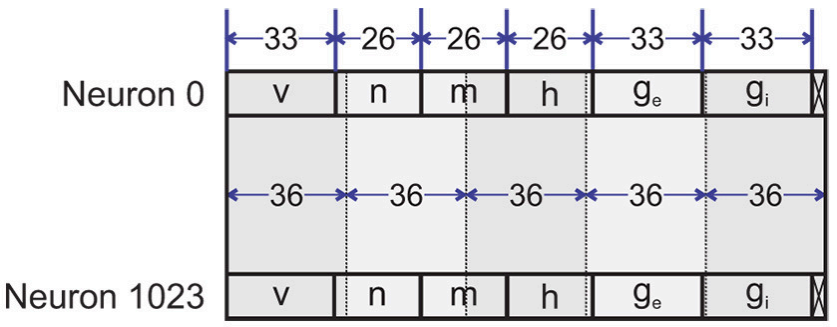
Layout of memory banks. Shaded boxes show the 1K×36 block memories.

As we said above, we exploit 33 bits (Q9.24) for each variable, but the gating variables only vary from 0 to 1. Therefore, we do not require nine bits for the integer part. As a result, we employ the Q9.24 format for *v*, *g*_*e*_, *g*_*i*_ and a reduced one for *m*, *n*, *h*. In the example of this section, we employ four memory banks called B0-B3. Each bank contains 1,024 words of 177 bits (Figure 8). Therefore, the pipeline requires 1024 clock cycles to complete an iteration. Each word stores the state variables including membrane voltage, three gating variables and two synaptic conductances for inhibitory and excitatory synapses. While the first three banks (B0-B2) store the states of excitatory neurons, the fourth bank (B3) holds the inhibitory neurons' states.

A 10-bit counter is used to address neurons' states stored in memory banks. In the first clock cycle, this neuron counter is an all-zero vector that addresses the 0th, 1024th, 2048th, and 3072nd neurons' state variables in parallel. Next clock cycle increments the counter and the next four neurons' states are fetched. This process continues until reaching the end of the memory banks. When the states of all neurons are updated, one iteration is completed.

#### 2.6.2. Update synaptic conductance unit

The *update synaptic conductance* unit is used to update inhibitory and excitatory synaptic conductances (*g*_*i*_ and *g*_*e*_ respectively) of the current neuron, according to presynaptic activities generated in the previous iteration. In order to update inhibitory and excitatory synaptic conductances, we should answer these two questions:

1. Which neurons have fired in the previous iteration?
2. Which neurons are the presynaptic neurons of the current neuron?

To describe the major contributions of the work, we will try to answer the above questions, along with other parts of the USC, in following subsections.

##### 2.6.2.1. Spike vector

To answer the first question, we use a 4096-bit spike vector (SV), in which 1s and 0s indicate if the spikes have occurred. For example, a 1 in the column 3165 of SV shows that the neuron 3165 located in B3 (so it is an inhibitory neuron) has fired in the previous iteration. This vector has been partially generated in WB stages of all four cores. At the end of each iteration (1024 clock cycles), these partial SVs, called SV0-SV3, are merged together to form the next iteration SV. A partial SV is a 1024-bit FIFO filled with the output of the comparator next to the UNS. This comparator compares *v*_*old*_ and *v*_*new*_ with a threshold value to determine the state of firing. If a neuron fires, a 1 enqueues to the FIFO, otherwise a 0 enqueues. After 1024 clock cycles, at the end of one iteration, all partial SVs are filled with the corresponding firing activities.

##### 2.6.2.2. Permutation vector

To answer the second question, we require a random 4096×4096 binary connectivity matrix. Each row of this matrix (here it is called the connectivity vector) shows the presynaptic neurons of the neuron associated with that row. Storing this matrix needs a memory with the size of 16Mb. Although this size of memory is not a large one for modern memory chips, it is still large for a mid-range FPGA with a few megabits of internal memory. For example, the most powerful FPGA of Artix®-7 family has only 13Mb of block memory. To overcome this shortcoming, it seems that there are only two choices. We have to either switch to a high-performance FPGA and pay its cost or use an external memory chip.

The problem still persists even if we choose the highest performance FPGAs. XCVU190 is a good example that with 132.9Mb on-chip memory has the highest amount of memory in a Virtex®-7 family. This device supports a connectivity matrix for just about 11.5k neurons. A better solution is to switch to external memories instead of internal ones. This solution eliminates the limitation of internal memory capacity but imposes a new constraint, which is the bandwidth of memory interface. The limited bandwidth dramatically increases the number of clock cycles needed to fetch a complete row of connectivity matrix. More cycles result in more simulation time. In the following paragraphs, we are going to review methods that address these problems. In the last part of this subsection, we introduce our alternative novel solution.

The first method includes techniques used to compress sparse matrices (Navabi, 2011). These algorithms reduce the size of the matrix, but they are inherently sensitive to the density of the matrix. The density of the connectivity matrix (which is defined as one minus sparsity and represents the probability of existing a connection between every two random neurons) is often assumed to be below 20%. A connectivity matrix with a lower density causes a more compressed matrix. Every density requires a specific hardware to decode the compressed matrix. Therefore, it is impossible to design an optimized unique hardware that supports all ranges of density values. In the search for a low cost and fast alternative method, we attempt to generate the rows of this matrix cycle by cycle instead of storing the whole connectivity matrix.

Generating a random binary vector with a specific sparsity to represent a row of the connectivity matrix is more complicated than it seems. There are several ways to generate random binary vectors using a pseudo-random number generator (PRNG). Some techniques use linear recurrence such as a linear congruential generator (LCG) (L'Ecuyer and Panneton, 2005). Improving the randomness is achieved by introducing modern algorithms such as linear feedback shift register (LFSR). The LFSR is the most popular technique used in the hardware implementation to make a binary random sequence (Golomb, 1981). Some algorithms are based on chaotic iterations and offer a higher degree of randomness (González and Pino, 1999). Furthermore, cryptographic algorithms can be used as a source of randomness in some applications (Petit et al., 2008). Due to the complexity of encryption and decryption processes, this technique is usually utilized when a cryptographic hardware accelerator is available. Finally, there is a hardware-friendly PRNG which is based on cellular automata. We can use 1D or 2D cellular automata (CA) or their hybrid forms to generate a PRNG sequence with an acceptable degree of randomness (Matsumoto, 1998; Shackleford et al., 2002; Comer et al., 2012). However, many papers have been published to investigate the CA-based PRNG from the randomness and hardware requirements points of views. A simple 1D CA rule, called Rule-30, results in satisfactory randomness as well as reasonable hardware resources. Unfortunately, binary vectors that are generated using PRNG techniques suffer from a drawback in our application: they have almost an equal number of ones and zeros. It means that they have a density of nearly 50%. As mentioned before, the density of a connectivity matrix often varies from network to network but remains below 0.2. To create a vector with a density different from 50%, it is possible to combine several independent pseudorandom vectors using bitwise logical operations. Although this technique is a practical approach, it has two major drawbacks. It is an inflexible approach which means that it cannot be used for generating a vector with arbitrary density. Furthermore, to generate a sparse vector, we require to compute the bitwise AND of several independent vectors which increases both the resource usage and latency.

To generate a random sequence with an arbitrary density, we should employ 4096 different PRNGs (for example Rule-30 CA with a minimum period of 4096). Then we compare their outputs with a threshold. The output of the comparator is an entry of the connectivity vector. Such a technique is not a good one from the resource usage point of view.The resource sharing can be employed to reduce the hardware cost at the expense of more clock cycles. Consequently, generating a connectivity vector using the above-mentioned approach suffers from either high resource usage or high latency.

**Figure 9:**
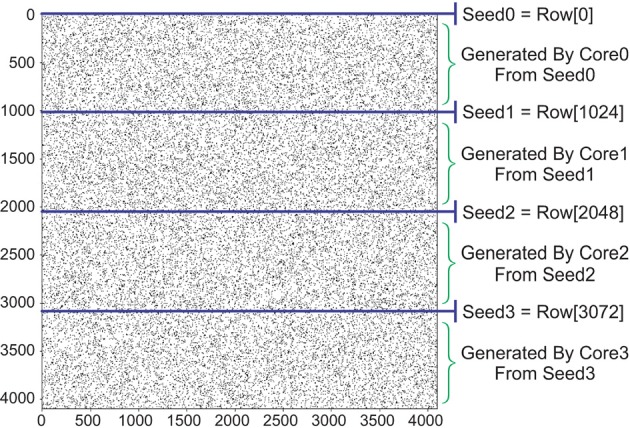
Generating a connectivity matrix using permutation matrix. Initial seed (Seed0) has a predfeined density. Next rows of the connectivity matrix are unique reordered versions of Seed0 using a 4096-cycle permutation matrix.

In this paper, we propose a low cost permutation-based technique to generate the connectivity vectors. This technique reduces the hardware cost to only one Flip-Flop for each element of the connectivity vector and some routing overhead. Our proposed algorithm starts with generating seeds, called here Seed0 to Seed3 (Figure 9). The following steps should be taken to generate the seeds for 4096 neurons:

1. Select an arbitrary value for the density of the connectivity matrix.
2. Generate an offline random connectivity vector with the above density using a software such as MATLAB. We call this vector Seed0 in later steps.
3. Find a 4096×4096 permutation matrix with 4096-cycle. We use python SymPy and NumPy packages.
4. Extend Seed0 for remaining 4095 rows using the above permutation matrix. We now have our desired connectivity matrix.
5. Split connectivity matrix into four 1024×4096 matrices. The first row of each submatrix is called Seed0, Seed1, Seed2, and Seed3 respectively.

Now inside the FPGA, we wire 4096 Flip-Flops together with the pattern of the above permutation matrix. Each core needs one of these units as its local PV register stated in Figure 7 **(Top)**. We assign each seed to its corresponding core and store it in the PV register as its initial value. This register in each clock cycle provides a new connectivity vector.

##### 2.6.2.3. Approximate counters

The content of SVs and PVs are the probable spikes and the connectivity vectors, respectively. Performing bitwise AND operation between these 4096-bit vectors give us another 4096-bit vector. Each “1” entry in this vector signifies a presynaptic neuron of the current neuron that has fired in the previous iteration. As mentioned earlier, the first 3072 bits belong to excitatory neurons, and the remainder of bits are representatives of inhibitory neurons. Therefore, we need two different counters to compute the impact of inhibitory and excitatory synapses on the current neuron. The upper and lower counters in Figure 7 **(Top)** are responsible for calculating coefficients of *w*_*e*_ and *w*_*i*_ respectively. In exact adders, we require 10 and 12 bits to represent the addition of 1024 and 3072 bits. Furthermore, we need a huge tree of full and half adders to implement such a counter. The exact counters are necessary for simulating fully connected networks, but there is a different story for the sparse connectivity matrix. Suppose that the density of the connectivity vector (PV) is below 20% (that is true in many cases). Performing AND operation with SV, which itself is a sparse vector, reduces the density of the outcome even more. Our simulation results show that the sparsity of AND(SV,PV) is always less than 2%. We can employ this sparsity to design an approximate counter with less hardware cost than the exact counterpart. Note that we cannot utilize approximate counters for fully connected networks. Instead, we have to use exact counter in these cases.

In an exact counter, we must employ two huge adder trees with 3072 and 1024 inputs to produce (3072:12] and (1024:10] exact counters respectively. The size of the adder tree progressively increases with the number of inputs. To reduce the hardware resources, we propose a novel two-level approximate adder. In the first level, we put some saturated counters to reduce the number of inputs to the next level. In the second level, we put an exact adder tree (see Figure S1 for more details). Table 3 compares the exact counter with several approximate ones from the resource usage and latency points of views. We evaluate the accuracy by putting the corresponding type of adder in our network hardware and comparing it with the results extracted from the network with an exact adder. Our simulations show that the approximation with the (64:2] saturated counter reach the accuracy of the exact model. Meanwhile, it consumes by far lower LUTs than the exact counter.

**Table 3:** A comparison between exact and approximate counters from the resource consumption and the latency points of views.

**Type**	**No. LUTs**	**Latency (nS)**
Exact	5777	14.907
(6:1]	1454	12.167
(8:2]	3532	13.938
(16:2]	3092	12.938
(32:2]	3068	12.186
(64:2]	3020	11.85
(128:2]	2460	9.916

*Both exact and shaded rows have equal accuracies for densities less than or equal to 20%*.

#### 2.6.3. Deep pipelining

Up to now, we have used a simple 3-stage pipeline to describe the functionality of our architecture in detail. Improving the performance could be achieved by adding more pipeline stages. Adding four levels of pipeline registers makes a 7-stage pipeline that introduces a higher clock frequency (see Figure S2 for more details). Fortunately, DSP48E1 offers internal pipeline registers. These internal registers can be used as the pipeline registers to save the FPGA fabric Flip-Flops and reduce the routing overhead.

## 3. Results

To demonstrate the capabilities of our proposed architecture, we use Verilog hardware description language to describe the example explained in section 2.6; that is, a network made of 4 cores and 4096 neurons. The Nexys Video board from Digilent is employed to implement the network. We organize the results as follows: First, the possibility of generating various network activities using this hardware is shown. Next, the results of the proposed hardware implementation are compared with those of the software model. Finally, we report the resource utilization and the hardware performance.

**Figure 10:**
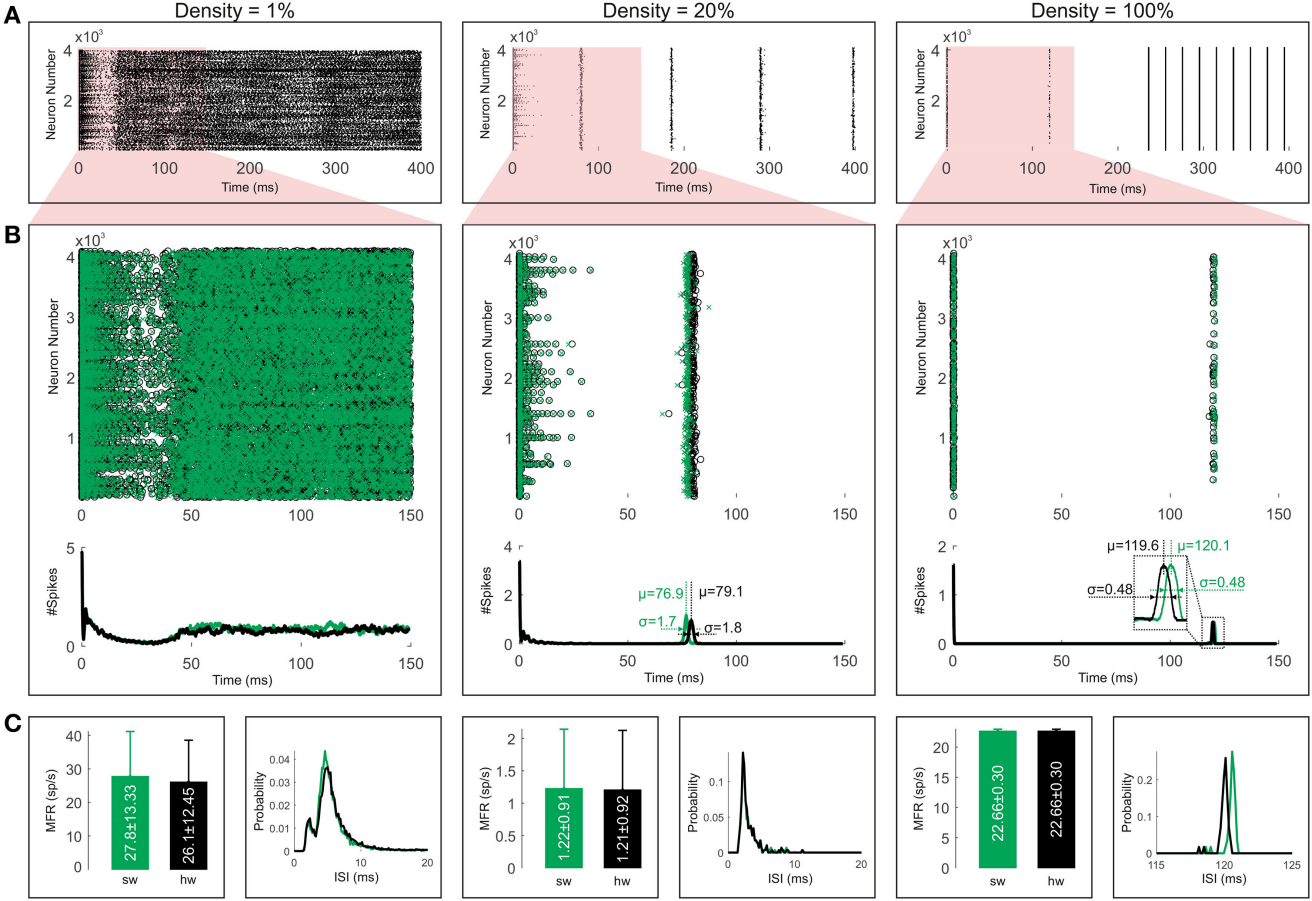
Comparing simulation results for both software (green) and hardware (black) implementations. **(A)** The raster plots are shown for three different sparsities of the network. **(B)** We focus on the first 150 ms to draw a comparison between the software and the hardware simulations. Moreover, the corresponding population activities are illustrated. **(C)** The bar and the line graphs are utilized to show the mean firing rates (MFRs) and the ISI distributions respectively. Error bars represent standard deviations.

For comparing the effects of the connection probability or the sparseness of the network on the network behavior, we show the spiking activities with different connection probabilities in Figure 10A. This figure illustrates the network activity for three different connection densities, including 1, 20, and 100%. We employ the exact hardware for simulating the fully connected network and the approximate one for the sparse networks. The network activities show irregular spiking for the density of 1% and synchronous spiking for the denser connections. The raster plots show similar behavior to many papers in the term of the relationship between sparseness and synchrony of the network activity (e.g., Börgers and Kopell, 2003; Uhlhaas et al., 2009).

The validation of the hardware simulation is carried out by comparing the raster plots with their software equivalents. Figure 10B illustrates the first 150ms of the raster plots and their corresponding population activities. To smooth out the population activities, we use a simple moving average with a window length of 128 iterations (1 ms). For the 1% density, the population activity of the hardware model matches the software one for about the first 50 ms whereas the network starts to display chaotic behavior after that. Since the chaotic systems are highly sensitive to initial conditions, slight differences between the values of the variables for the hardware and software models can result in significant differences in the later states. It, therefore, is reasonable to expect two chaotic patterns that do not necessarily agree. Different number representations have different precisions (roundoff errors) which affect the evolution of the network. Chaotic systems with different number representations, therefore, meet completely different destinies even for the double and the single precision floating-point representations in software simulations (see Figure S3 for more details).

While the propagation of uncertainties originated from the fixed-point number representation and PWL approximations of non-linear functions causes a different chaotic behavior in the sparsest case, it results in slight jitters in firing times of neurons in the case of two other network connectivities. To investigate the average jitter values, we provide a comparison of population activities in the hardware model and the software model for the first synchronous firing columns which show the highest jitters in these raster plots. Regardless of leading or lagging, the hardware model shows average jitters of μ = 79.1*ms* − 76.9*ms* = 2.2*ms* and μ = 120.1*ms* − 119.6*ms* = 0.5*ms* for densities of 20% and 100% respectively. As it can be seen, the fully connected network has lower jitter value than the network with density of 20%. Overall, this analysis shows that the hardware simulations have close resemblances to the software results in terms of the spike timing.

Figure 10C compares the raster plots from the ISI distributions and the mean firing rates (MFR) criteria points of views. The line graphs show the histograms of the ISI values for the software and the hardware models (bin width equals 0.15 ms). For the sake of clarity, these graphs are confined to the range of the most probable ISIs. There are close similarities between the graphs for all connection densities. Quantitatively, their correlation coefficients are 0.99, 0.96, and 0.93 for the densities of 1, 20, and 100% respectively. Regarding the MFR, the bar graphs of Figure 10C illustrate the mean firing rates for the whole simulation time of Figure 10A. Although both software and hardware implementations show slight MFR differences for the two higher densities (20 and 100%), such differences are not statistically significant [*p* > 0.05, (*p*_*density*=20%_ = 0.5, *p*_*density*=100%_ = 0.9), paired sample *t*-test]. But for the density of 1%, this difference is statistically significant [*p* < 0.05, (*p*_*density*=1%_ = 1*e*−5), paired sample *t*-test] as expected for two different chaotic patterns (see Figure S3 for more details). In sum, our results show that the proposed approximation techniques and the used number system representations are accurate enough to capture the main patterns of activities in the network model and give a reasonably precise description of what happens in the software model.

To evaluate the proposed architecture from the performance and area points of views, we summarize the timing and utilization reports in Table 4. This table reveals two interesting points. First, the table makes a comparison between the resource utilization of the exact and the approximate implementations. As it can be seen, the approximate one consumes only 29130 LUTs, in contrast with 46045 LUTs used by the exact one. This value shows almost 37% reduction in the resource utilization. Secondly, a deeper pipeline results in higher performance. While single cycle implementation reaches the maximum frequency of 25.6 MHz for the approximate network, the pipeline implementation gets to the 71.4 MHz. The 7-stage pipeline makes a considerable gain in exchange for almost only 1000 registers. This small number of extra registers does not affect the final utilization. As a result, we always employ the pipeline hardware, exact one for the fully connected network and approximate one for the sparse network (density≤20%).

**Table 4:** Post place and route reports for a network with four cores.

	**Single Cycle**				**Pipeline**			
	**Exact**		**Approximate**		**Exact**		**Approximate**	
	**Timing (Frequency)**				**Timing (Frequency)**			
	**23.3 MHz**		**25.6 MHz**		**58.8 MHz**		**71.4 MHz**	
	**Resource**				**Resource**			
	**Used**	**(%)**	**Used**	**(%)**	**Used**	**(%)**	**Used**	**(%)**
LUT	46,045	34	29,130	21	46,045	34	29130	21
LUT-FF pairs	8,465	6	5,326	4	4,606	3	2810	2
Slice Registers	24,600	9	24,600	9	25,430	9	25430	9
DSP Blocks	280	37	280	37	280	37	280	37
BRAM	20	6	20	6	20	6	20	6

*While the Approximate columns show the results of the networks employed the two-level approximate counters, the networks in the Exact columns use exact single level counters*.

The number of cores per chip is limited by the most utilized logic resource, which is the DSP block with utilization of 37%. It means that for each core, we require a bit less than 10% of DSP blocks. If we tend to use the full capacity of our device, we will be only able to implement up to 10 cores per device (Table 4). As mentioned before, to achieve higher performances or larger networks, we could employ deeper or shallower core banks.

According to Table 5, for example, if we configure the memory banks in their shallower form (512×72), we only require three block memories per bank to provide the required 177 bits for storing membrane voltage, gating variables, and synaptic conductances. Furthermore, XC7A200T only contains 365 blocks of memories. Therefore, we could implement 365/3 = 121 banks in a device, but we should not forget that the number of DSP blocks limits the maximum number of cores to only 10. Thus, the maximum number of cores is bounded to the minimum number imposed by the memory or DSP blocks. It means that 5120 is the maximum number of neurons that could be implemented in this case. A single iteration for all neurons requires 512 clock cycles. As $dt=\frac{1}{128}ms$, a 1ms simulation requires 128 iterations. If we employ the approximate pipeline hardware with the frequency of 71.4MHz, this configuration shows almost a real-time speed or even faster if we partially fill the banks. Interestingly, switching to the 8k×4 configuration simply enlarges the network to 65536 neurons while it is only 15 times slower than real-time. For the example of this paper with 4096 neurons, the memory banks contain 1024 rows, therefore the simulation time is half real-time.

**Table 5:** Block memory configurations for both real-time and large-scale neuronal networks (time resolution = $\frac{1}{128}$ms).

**Type**	**Block memory configuration**	**No. BRAM/Bank**	**No. core**	**No. neurons**	**Performance (FPGA/real-time)**
Real-time	512×72	3=⌈177/72⌉	10	5120	0.94
Large-scale	8k×4	45=⌈177/4⌉	8	65536	15.09

## 4. Discussion and conclusion

The main purpose of this paper is to demonstrate that although the SNNs are compute-intensive and memory-intensive, it is possible to run either real-time or large-scale simulation using cost-optimized FPGAs. The hardware implementations of SNNs are utilized as accelerators not only in the field of computational neuroscience but also in the field of machine learning. They have much less energy consumption and much higher performance than their software equivalents. Using cost-optimized hardware enables more neuroscientists to test their hypotheses in a reasonable time. Furthermore, the SNNs have become increasingly popular in the field of machine learning. They are the third generation of neural networks (Maass, 1997) and can be an emergent computing paradigm, in contrast with traditional Von Neumann architecture. Our work makes the FPGA-based implementation of SNNs a proper candidate to bring intelligence to embedded cognitive systems.

In this work, we focus on an architecture to implement a randomly connected network of Hodking-Huxley neurons. Instead of utilizing dedicated weights for synapses, fixed synaptic weights are used to update the synaptic conductances. Nevertheless, these types of networks are widely used in the field of both neuroscience and machine learning. As an example, there is at least a population of randomly connected neurons in many neuronal networks and our architecture can be recruited in them too.

Introducing a novel method to update synaptic weights is our main contribution. We use a permutation-based technique to generate connectivity vectors on the fly and diminish the memory requirement for storing the whole connectivity matrix (section 2.6.2.2). In this method, connectivity vectors are generated by permuting an initial seed, with a given density, on each clock cycle. To avoid generating repetitive connectivity vectors, we select a permutation matrix with a cycle of length *N*, where *N* is the number of neurons. Although the randomness of the connectivity matrix generated by this method is not comparable with modern PRNGs, its advantages clearly outweigh this disadvantage.

In order to reduce the computational complexity of the neuronal network, we employ the approximation techniques in different parts of our design. First, a simple and general piecewise linear approach is used to approximate the inverse time constant and steady states of the gating variables (section 2.4). In this technique, we simply partition the entire range into equal intervals and approximate the resulting curves by the straight lines using the least square algorithm. Moreover, another approximation technique is used to build a faster and smaller adder that counts the presynaptic network activities for the case of a sparse network. This is a two-level adder that has the saturated counters in its first level (section 2.6.2.3). This technique reduces the resource usage almost 37% and increases the frequency almost 21% (Table 4). However, its accuracy is the same as the exact counter for the connectivity matrices that have a density smaller than 20%.

In a typical neuronal network, we require at least a few thousands of the neurons, and each consumes lots of logic resources. If we tend to implement a large-scale network, we will have to share the logic resources among a group of neurons. It adversely affects the overall throughput of the system. Therefore, a 7-stage pipeline is used to increase the throughput. Consequently, a large-scale and fast simulation of neuronal networks can be achieved by using both resource sharing and pipelining techniques simultaneously. Moreover, we can control the number of neurons per group to fulfill the requirements such as simulation speed and size of the network. In order to speed up the simulation time, a smaller group size is used. However, a larger one is exploited to enlarge the network size.

Our results confirm that our approximation techniques dramatically reduce both computational complexities and memory usage and make our architecture suitable for embedded applications, even though the most complex neuron model is used in our work. While some works exploit either Hodgkin-Huxley neuron model or its variants (Zhang et al., 2013; Smaragdos et al., 2014; Osorio, 2016; Yang et al., 2017), some other works utilize its reduced forms (Graas et al., 2004; Yaghini Bonabi et al., 2014). Meanwhile, the majority of researchers use simple neuron models such as Izhikevich or leaky integrate and fire (Cassidy et al., 2007; Soleimani et al., 2012; Ambroise et al., 2013; Furber et al., 2013; Cheung et al., 2016; Pani et al., 2017) to cope with the computational complexity of the HH model.

One of the most prominent digital implementations of a general-purpose simulator for SNNs is SpiNNaker (Furber et al., 2013). Each node is a chip that contains 18 identical ARM9 processors called cores. One of the cores is a monitor processor that performs system management tasks and another one is reserved for fault tolerance. Each node also contains a router, which is used to create the biggest NOC in the world. It could be configured to simulate both the LIF and the Izhikevich neuron models. Although the SpiNNaker naturally exhibits superior performance, our proposed architecture is still comparable from a few perspectives. While each custom node of the SpiNNaker could simulate 16K neurons with a time step of 1ms, our architecture is capable of simulating up to 65536 much more complex neurons with a 128 times smaller time step. Furthermore, contrary to the SpiNNaker, our design is implementable on a single FPGA chip and, thus, easily accessible for researchers.

A more affordable solution to build a general-purpose hardware simulator is FPGA. NeuroFlow (Cheung et al., 2016), as mentioned in Introduction, is a great project that uses HLS to implement a high-level Java code on a cluster of FPGAs. Moreover, its number representation is the floating-point which is more accurate than the fixed-point with the same word length. Advantages and disadvantages of the floating-point representations will be discussed in the following paragraphs. Furthermore, the designers of NeuroFlow have selected external DRAMs to store neuron parameters. Although this is inevitable when a large-scale network must be simulated, it is possible to store parameters in small BRAMs for some networks by designing an architecture with less memory usage. The latter approach is adopted in this paper.

An efficient hardware realization of the subthalamic nucleus–external globus pallidus oscillation system is introduced in Yang et al. (2017). They employ a variant of the HH model that includes various Calcium currents to reproduce required dynamics. In common with us, they approximate nonlinear functions of their neuron model using a PWL technique. Compared to our PWL approximation, while we select lengths of pieces as a power of two to better utilize block memories, they choose the slope values in that way to replace multipliers with shift operators. Moreover, they employ $\frac{1}{64}+\frac{1}{256}$ for the time step which is adequate for most applications, but it is coarser than ours. Finally, they use the same number representation as us. They use Q10.20 that is consistent with what has been found in our study (i.e., Q9.23).

A recent study proposes an FPGA implementation of 1440 Izhikevich neurons in a fully connected network (Pani et al., 2017). Although there are some similarities between their study and our proposed architecture such as scalability and modularity, a number of differences exist. While our architecture is designed in a way that supports arbitrary connection densities for a randomly connected network, their study only simulates fully connected circuits. Moreover, their work simulates 1440 neurons in real-time with a step size of 100μ*s* while our architecture is capable of simulating 5120 neurons in real-time with a smaller step size of 7.8μ*s* (see Table 5). If we select a step size of 100μ*s*, which is 12.8 times larger than its initial value, we can simulate 12.8×5120=65536 neurons in real-time.

In line with the idea of using PWL technique, a multiplier-less biologically inspired neuron model based on the Izhikevich simple model is demonstrated in Soleimani et al. (2012). Their neuron model utilizes the PWL method to approximate the quadratic nullcline of Izhikevich neuron model. Their proposed piecewise linear neuron model has a low hardware cost. However, because of the lack of resource sharing, their final network contains only 30 neurons. Moreover, their selected time step is almost eight times larger than ours. Although PWL methods for approximating nonlinear functions are widely used in the literature, some works focus on other realizations of nonlinear functions. For instance, in Yaghini Bonabi et al. (2014), the CORDIC is used to implement exponential functions of the HH neuron model. The CORDIC is more accurate than a PWL approximation at the cost of more resource usage. Moreover, their architecture, similar to Soleimani et al. (2012), lacks resource sharing. Therefore, their approach is capable of implementing up to only 150 independent reduced HH neurons. Their word length (Q10.22 fixed-point) appears to be consistent with our results and (Yang et al., 2017).

Unlike our paper, some authors implement conductance-based neuron models using the floating-point arithmetic, more stable numerical integration methods than the Euler method, or both. For example, Osorio (2016) presents an FPGA architecture for applying the 4th order Runge-Kutta (RK4) rather than the Euler method to the HH model. RK4 is more stable and accurate than a first-order numerical procedure such as the Euler method. However, its hardware implementation consumes far more hardware resources. Moreover, their number representation is the double-precision floating-point. The author generates customized floating-point cores for the required arithmetic functions using the FloPoCo. Combining double-precision floating-point representation with RK4 results in the most similar FPGA implementation to the software implementation. Meanwhile, this architecture consumes more resources and burns more power than other architectures. In another work, (Smaragdos et al., 2014) accelerates an extended HH model for Inferior-Olive neurons. The VIVADO HLS is utilized to implement a network with at most 14,400 neurons using single precision floating-point computations in a Virtex 7 FPGA. In line with the previous study, (Zhang et al., 2013) introduces an HH neuroprocessor which is capable of generating complex spiking patterns using single-precision floating-point numbers. The authors combine their customized accelerators (neuroprocessors) with a LEON3 processor and an advanced microcontroller bus architecture (AMBA) to build a system on chip (SOC). LEON3 configures the neuroprocessors and dispatches spikes between cells. Overall, some authors investigate more precise number formats and numerical procedures to guarantee the convergence for complex neuron models and neuronal networks. For a given word length, the floating-point representations show higher precision than fixed-point for simulating spiking neural networks. A higher precision results in lower jitter value and may be necessary in tasks that require precise spike timing. Despite this fact, the majority of studies pay more attention to fixed-point representations due to the complexities of designing floating-point modules and their resource requirements.

In this work, we illustrate how the combination of some techniques can provide an embedded implementation of a randomly connected large-scale SNN. This work is a part of our long-term goal. That is, our desire to bring the intelligence to the embedded world. As an example of a possible future application, we will consider the possibility of employing our architecture as a reservoir of a liquid state machine (Maass, 2011).

## Author contributions

KA-S and SS defined the methods. KA-S and A-HV developed the theoretical framework. KA-S and BA performed the simulations and implemented the design on the FPGA. All authors discussed the results and contributed to the final manuscript.

### Conflict of interest statement

The authors declare that the research was conducted in the absence of any commercial or financial relationships that could be construed as a potential conflict of interest.
