# Supplementary material for: A Scalable FPGA Architecture for Randomly Connected Networks of Hodgkin-Huxley Neurons
Source: Front Neurosci. 2018 Oct 9;12:698. doi: 10.3389/fnins.2018.00698 (PMC6190648; doi:10.3389/fnins.2018.00698)
Supplement: Supplementary file 1 [file Image_1.pdf]

Supplementary Material:
A Scalable FPGA Architecture for Randomly Connected Networks of Hodgkin-Huxley Neurons

1 FIGURES

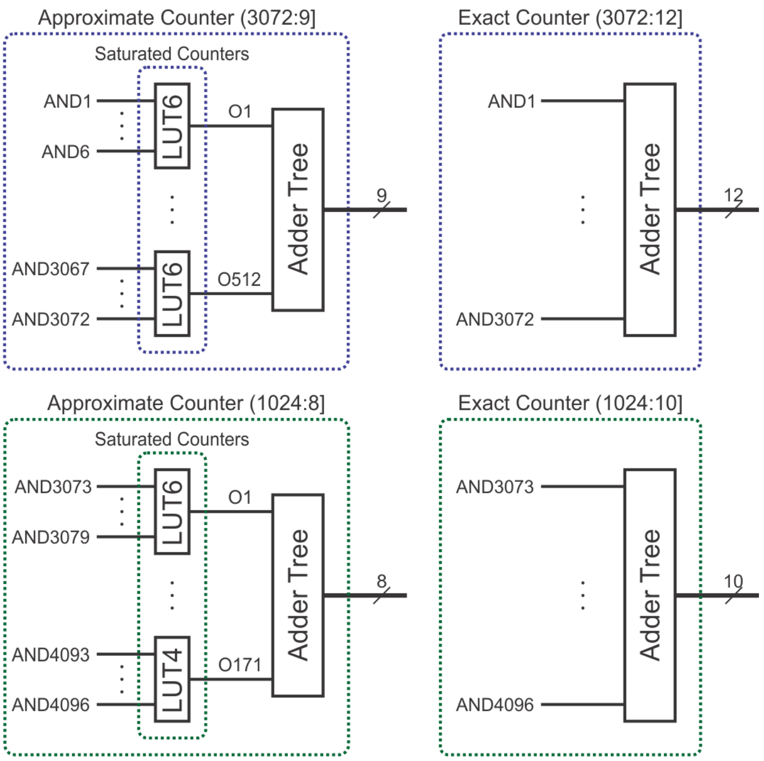

Figure S1. Approximate versus exact counters for inhibitory and excitatory banks. Approximate counters utilize a set of saturated counters composed of FPGA LUTs to reduce the size of exact adder trees. This technique reduces the size of the adder trees from 3072:12 and 1024:10 to 512:9 and 171:8 for excitatory and inhibitory banks respectively for the case where saturation counters are configured to (6:1]. The (n:1] counters only indicate the presence of a '1' on their input string, regardless of its position.

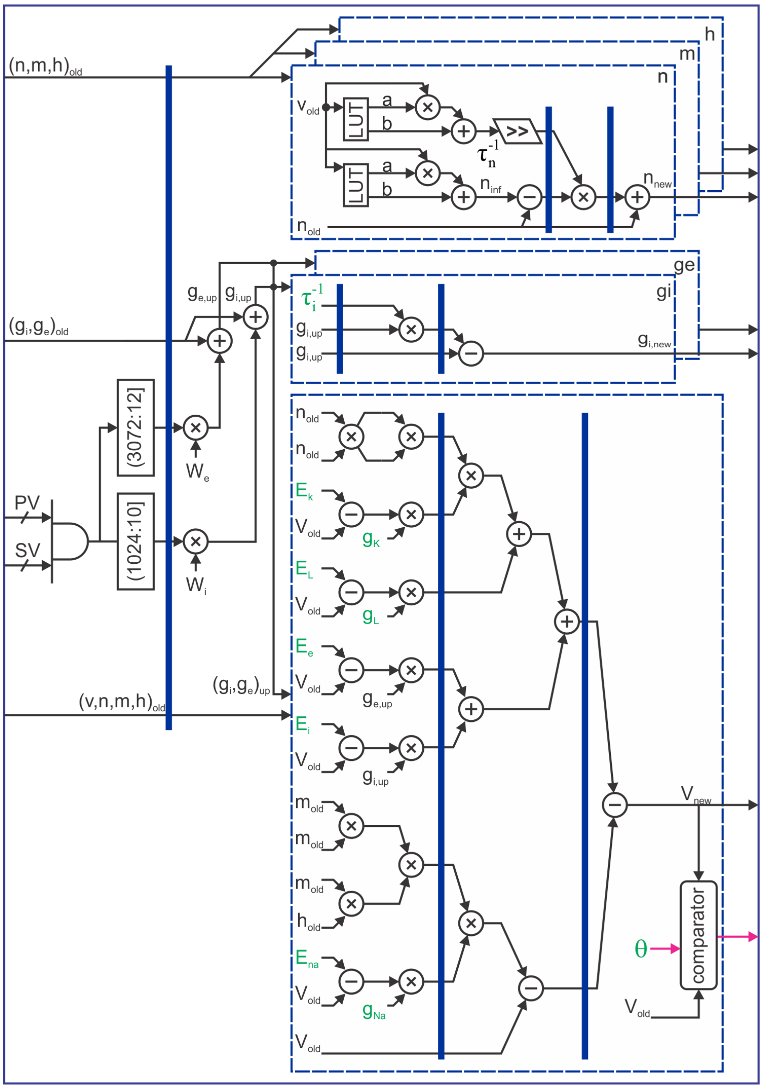

Figure S2. Data flow graph (DFG) of the USC and UNS parts of the design. Vertical blue lines show locations of registers for a sample pipelining of the datapath.

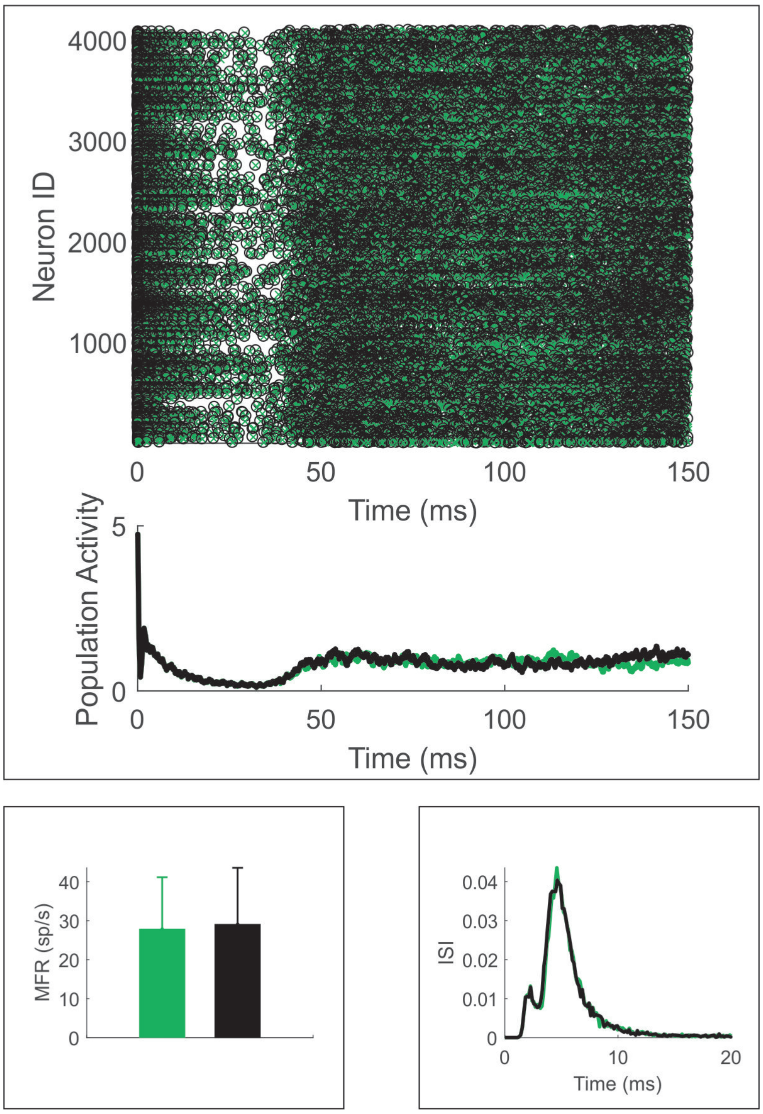

Figure S3. Comparing simulation results for both double precision (green) and single precision (black) software implementations. (a) The raster plots of the network are shown for the density of 1% where the chaotic behavior is observed. (b) We focus on the first 150ms to draw a comparison between both precisions. The corresponding population activities are shown. As it clearly shows, similar to what is happened in our hardware model, two population activities do not match after 50 ms, when the network slips into chaos. (c) Bar and line graphs are utilized to show the mean firing rate (MFR) and the ISI distributions respectively. In this case, like the comparison between hardware and double precision software implementations, MFRs have significant differences ($p < 0.05$, ($p_{density=1\%} = 2e - 3$), paired sample t-test) that is an inevitable consequence of chaotic systems. The similarity between ISI distributions is quantitatively verified by the correlation coefficient value that is 0.97.
